# Predicting ecosystem components in the Gulf of Mexico and their responses to climate variability with a dynamic Bayesian network model

**DOI:** 10.1371/journal.pone.0209257

**Published:** 2019-01-23

**Authors:** Neda Trifonova, Mandy Karnauskas, Christopher Kelble

**Affiliations:** 1 University of Miami Cooperative Institute for Marine and Atmospheric Studies CIMAS, Miami, United States of America; 2 NOAA Atlantic Oceanographic and Meteorological Laboratory, Miami, United States of America; 3 NOAA Southeast Fisheries Science Center National Marine Fisheries Service, Miami, United States of America; University of Pittsburgh, UNITED STATES

## Abstract

The Gulf of Mexico is an ecologically and economically important marine ecosystem that is affected by a variety of natural and anthropogenic pressures. These complex and interacting pressures, together with the dynamic environment of the Gulf, present challenges for the effective management of its resources. The recent adoption of Bayesian networks to ecology allows for the discovery and quantification of complex interactions from data after making only a few assumptions about observations of the system. In this study, we apply Bayesian network models, with different levels of structural complexity and a varying number of hidden variables to account for uncertainty when modeling ecosystem dynamics. From these models, we predict focal ecosystem components within the Gulf of Mexico. The predictive ability of the models varied with their structure. The model that performed best was parameterized through data-driven learning techniques and accounted for multiple ecosystem components’ associations and their interactions with human and natural pressures over time. Then, we altered sea surface temperature in the best performing model to explore the response of different ecosystem components to increased temperature. The magnitude and even direction of predicted responses varied by ecosystem components due to heterogeneity in driving factors and their spatial overlap. Our findings suggest that due to varying components’ sensitivity to drivers, changes in temperature will potentially lead to trade-offs in terms of population productivity. We were able to discover meaningful interactions between ecosystem components and their environment and show how sensitive these relationships are to climate perturbations, which increases our understanding of the potential future response of the system to increasing temperature. Our findings demonstrate that accounting for additional sources of variation, by incorporating multiple interactions and pressures in the model layout, has the potential for gaining deeper insights into the structure and dynamics of ecosystems.

## Introduction

The Gulf of Mexico (GoM) is an ecologically and economically productive ecosystem, generating over 2.6 million metric tons of commercial fishery landings in 2016 and supporting nearly half the recreational fishing catch in the entire United States [1]. The ecosystem is exposed to a number of pressures including: coastal development, oil spills, hurricanes and major hypoxia formation on the continental shelf of Louisiana and Texas. Many of the primary human activities that affect coastal and marine environments occur in the GoM: extraction of living resources, transport and shipping, fossil fuel energy, coastal and land-based infrastructure and industry [2]. These activities result in a number of pressures being placed upon the GoM ecosystem that are both exogenic unmanaged pressures and endogenic managed pressures. These pressures occur against a background of more chronic stressors related to climate change, which include gradual changes in temperature and ocean acidification. The delineation of pressures into exogenic and endogenic requires resource managers and scientists to define those that are manageable within the system from those pressures that cannot be managed, but whose consequences must be accounted for, such as temperature changes due to climate change [2]. Analyses and models have shown that the GoM is already experiencing increasing sea surface temperatures and that these increases are expected to continue and intensify throughout the 21st century [3]; however, the changes are relatively muted in comparison to other parts of the globe [4]. In other oceanic regions, the effects of long-term, temperature-driven changes on fish populations and fisheries are already apparent and well-documented. For example, in the Northwest Atlantic, there have been significant poleward shifts in major commercial species and their associated landings [5]. In the GoM, a northern land mass prevents species from making poleward shifts, and thus, species must either move deeper, die out, or adapt when temperatures increase beyond their tolerance levels. Indeed, shifts in depth distributions and the tropicalization of fish communities have already been documented in the GoM [6–7]. However, effects from increasing temperatures are not as well documented or understood in the GoM as in other marine ecosystems. The lack of documented effects from climate change results in the GoM receiving less attention for understanding ecosystem and fishery responses to climate change. Moreover, the unique geography of the GoM, its high biological diversity, and the range of complex and interacting pressures make it challenging to predict the overall impact of climate change on the ecosystem. These all highlight the need to begin building a knowledge base for predicting how the GoM will respond to the impending effects of climate change. In situations, where all of the pertinent mechanistic relationships between drivers and responses cannot possibly be understood, it is valuable to employ data-driven approaches. Significant progress has been made in developing ecological models that use traditional statistical approaches to understand the relationships between a number of interacting variables [8], and more recently, the number of all-inclusive models from physics through to higher level species is increasing [9].

However, when these models are parameterized using data, it is usually assumed that the ecosystems are in a steady state. Thus, the underlying relationships that give rise to the data are assumed to not be changing. This assumption is incorrect in many cases, as it is known that ecosystem structure and function can change even over relatively short time scales [10]. Further, it is possible that such changes are driven by unobserved variables, i.e. ecosystem variables that we do not have data on, and being able to account for such signals is difficult but crucial for the protection and sustainable use of ecosystems. Indeed, it is recommended that forecasting models develop richer non-mechanistic appreciation of ecological interactions and predict beyond the range of observed values in combination with scenarios to express uncertainties and test policies [11]. Predicting species response to ecosystem changes is challenging because of the natural variation in observations and uncertainty in potential associations. However, machine learning techniques have been proposed to be an appropriate approach with desired properties to address uncertainty in ecosystem prediction [12]. Recently, Bayesian networks (BNs) have become a popular method in the area of biology that is capable of inferring network structures, while capturing nonlinear, dynamic and arbitrary combinatorial relationships [13]. It has been shown that such probabilistic methods can provide estimates of the uncertainty associated with predictions and recover complex, spatially varying interactions from collected field data [14]. The way BNs manage uncertainties is through developing alternative model structures and accounting for these uncertainties in the distribution of probabilities across the BN nodes and their states [15]. Given a graphical structure, BNs naturally perform prediction using inference that allows us to ask “what if?” type questions of the data. For example, one could ask, what is the probability of seeing a change in zooplankton, given that we have observed a change in the distribution of temperature and/or primary productivity? Formerly, BNs have been applied to reveal gene regulatory networks using gene microarray data [16]. Other BN uses include weather prediction, medical diagnosis and image processing [17–19]. BNs have proven quite useful to predict outcomes for situations where we do not fully understand or have data on the underlying mechanisms. Due to the flexibility in application and lack of dependence on a *priori* assumptions about mechanistic relationships, BNs are becoming increasingly popular for modeling uncertain and complex domains such as ecosystems and environmental management [20–22]. In an environmental study context, BNs represent probabilistic dependencies among species and ecosystem factors that influence the variables likelihood in an intuitive, graphic form [23]. Therefore, expertise can utilize a quantitative indication of the range of possible scenarios consistent with the data to give strategic advice on potential ecosystem response. The use of BN methodology facilitates the communication of modeling results and the representation of a variety of perspectives as a means of modeling likelihoods of natural and anthropogenic effects [24]. In this study, we apply BN approaches to examine the potential responses of different ecosystem components (i.e. variables in the analysis that represent either physical processes thought to be important drivers of the ecology, or biological components; specifically, estimates of abundance or population productivity) to increases in temperature within the GoM marine ecosystem. Resource management in the GoM requires an understanding of how key species will respond to likely temperature increases to enable effective future management of the species and to prepare fishermen and their communities for likely ecosystem changes. First, we evaluate the predictive capabilities of a variety of dynamic BNs which reflect the different hypothesis of the GoM system and consider varying levels of complexity. In these models, we incorporate a number of hidden variables to account for uncertainty and any unmeasured effects. This includes a novel approach for modeling the Gulf ecosystem dynamics by accounting for multiple physical and biological associations and their changes over time. We examine the models’ accuracy in terms of their ability to reproduce observations of ecosystem components of interest. Then, the best performing model is applied to investigate how the GoM ecosystem will respond to increasing temperature, specifically examining whether ecosystem components are likely to be negatively or positively affected by temperature increases. Through the developed temperature scenarios, we explore the components trends in response to climate variation. Thus, we aim to gain further understanding of the underlying ecological mechanisms and the consequences on the different ecosystem components once some of these mechanisms are perturbed, which is essential in terms of providing advice on potential response of the ecosystem to the full suite of pressures. Here, by testing short-term predictability and variability on this marine ecosystem, we further build confidence in our ability to examine potential long-term impacts of environmental change that could inform strategies for coping with and adapting to climate change and variability.

## Methods

### Data

Because the method is computationally intensive, and also to reduce the risk of introducing spurious relationships in our predictions, we included only the drivers for which defensible linkages to the biological dynamics could be made. The ecosystem components (or variables) examined in this study were selected by carefully balancing considerations regarding management linkages, data availability, statistical robustness, and representation in spatial and temporal dimensions, following up on the 2017 Ecosystem Status Report Update for the Gulf of Mexico [25]. The major categories of ecosystem components included in the analysis were climate drivers, physiochemical ecosystem pressures, lower trophic levels, recruitment deviation estimates for economically important species and population estimate for a piscivorous bird (Table 1). We include an index of the Atlantic Multidecadal Oscillation (AMO), which is influential in structuring dynamics at both ecosystem- wide and species-specific scales [26–27]. Like other modes of variability (e.g. El Nin˜o Southern Oscillation), the AMO has impacts on a large geographic scale via—atmospheric teleconnections, and has been hypothesized to have an influence on a range of North Atlantic fisheries and ecosystems [28–29]. Thus, multiple lines of evidence suggest that the AMO may be the underlying driving force for many observed changes within the Gulf of Mexico ecosystem [30–31]. We did not include a separate ecosystem component for hurricanes, because they are a pulse disturbance system and this study is focused upon broad-geographic scale long-term presses on the Gulf of Mexico ecosystem.

**Table 1:** Summary of data.

CATEGORY	ECOSYSTEM COMPONENT	EXPLANATION	SOURCE
Climate	AMO	Atlantic Multidecadal Oscillation	NOAA’s Earth System Research Laboratory
Climate	SST TX	Sea surface temperature of the southern Texas shelf	Adapted from [25].
Climate	SST LA	Sea surface temperature along the Louisiana shelf	Adapted from [25].
Climate	SST FL	Sea surface temperature from the west Florida shelf	Adapted from [25].
Physiochemical	TN	Total nitrogen for the Mississippi-Atchafalaya river basin	US Geological Survey. Available: https://cida.usgs.gov/quality/rivers/coastal
Physiochemical	TP	Total phosphorus for the Mississippi-Atchafalaya river basin	US Geological Survey. Available: https://cida.usgs.gov/quality/rivers/coastal
Physiochemical	Summer LA DO	Bottom water dissolved oxygen concentration for the Louisiana coastal shelf in summer (5-110m depth)	Southeast Area Monitoring and Assessment Program (SEAMAP) trawl and hydrographic survey
Physiochemical	Summer TX DO	Bottom water dissolved oxygen concentration for the Texas coastal shelf in summer (5-110m depth)	SEAMAP
Physiochemical	Fall LA DO	Bottom water dissolved oxygen concentration for the Louisiana coastal shelf in fall (5-110m depth)	SEAMAP
Physiochemical	Fall TX DO	Bottom water dissolved oxygen concentration for the Texas coastal shelf in fall (5-110m depth	SEAMAP
Primary production	NPP	Net primary production for the northern Gulf area above 25° N latitude	Moderate Resolution Imaging Spectrometer (MODIS) observations. Adapted from [25].
Population estimate	Spring zooplankton	Zooplankton biovolume (ml m^-3^) calculated for spring survey (open ocean from the shelf break to the extent of the U.S. Exclusive Economic Zone)	SEAMAP. Adapted from [25].
Population estimate	Fall zooplankton	Zooplankton biovolume (ml m^-3^) calculated for fall survey (from nearshore to outer continental shelf)	SEAMAP. Adapted from [25].
Stock productivity	Pink shrimp	Recruitment deviation	Stock Assessment
Stock productivity	Brown shrimp	Recruitment deviation	Stock Assessment
Stock productivity	White shrimp	Recruitment deviation	Stock Assessment
Stock productivity	Menhaden	Recruitment deviation	SEDAR 27A 2015 Stock Assessment
Stock productivity	Cobia	Recruitment deviation	SEDA 28 2013 Stock Assessment
Stock productivity	Gag grouper	Recruitment deviation	SEDAR 33 2016 Stock Assessment
Stock productivity	Red grouper	Recruitment deviation	SEDAR 42 2015 Stock Assessment
Stock productivity	Red snapper	Recruitment deviation	SEDAR 31 2014 Stock Assessment
Stock productivity	Spanish mackerel	Recruitment deviation	SEDAR 28 2013 Stock Assessment
Stock productivity	Greater amberjack	Recruitment deviation	SEDAR 33 2016 Stock Assessment
Stock productivity	King mackerel	Recruitment deviation	SEDAR 38 2014 Stock Assessment
Stock productivity	Gray triggerfish	Recruitment deviation	SEDAR 43 2015 Stock Assessment
Stock productivity	Vermillion snapper	Recruitment deviation	SEDAR 45 2016 Stock Assessment
Stock productivity	Tilefish	Recruitment deviation	SEDAR 25 2016 Stock Assessment
Population estimate	Brown pelican	Index of abundance for pelican in the coastal GoM	Cornell Lab of Ornithology’s eBird. Adapted from [25].

**Fig 1:**
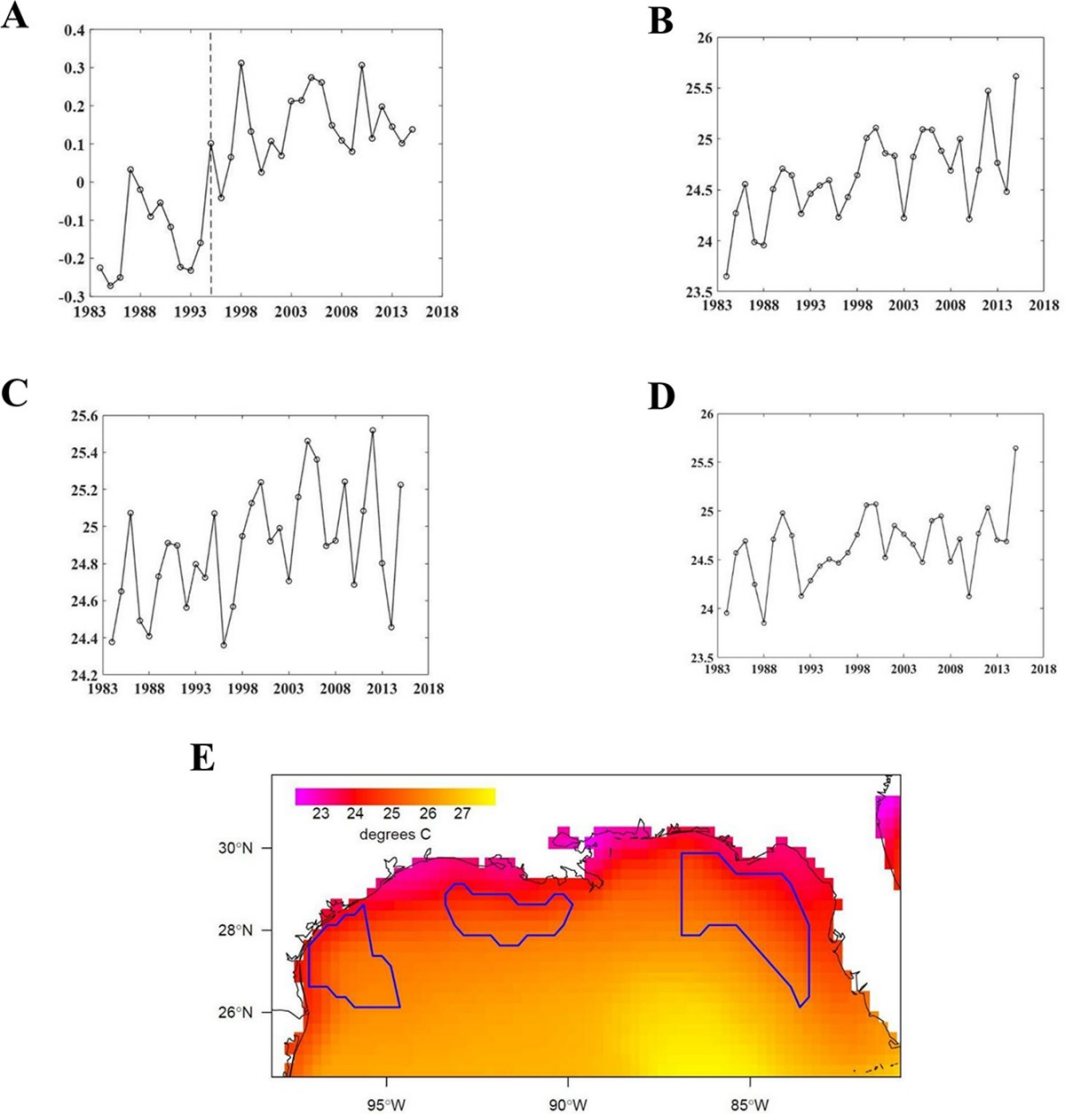
The Atlantic Multidecadal Oscillation index and sea surface temperature values. (A) Annual averages of the Atlantic Multidecadal Oscillation index. The vertical line indicates the beginning of the warm phase in 1995. (B) Annual averages of sea surface temperatures over the Texas shelf (SST TX), (C) Louisiana shelf (SST LA) and (D) west Florida shelf (SST FL). (E) Map of the SST regions, from left to right: SST TX, SST LA and SST FL.

For the climate drivers, we used yearly averages from the monthly unsmoothed AMO index (Fig 1A) and yearly sea surface temperature (SST) values, calculated for select regions within the GoM. The mean offshore SST was found to be one of the most significant indicators in terms of the mid-1990s ecosystem reorganization, and is available at fine spatial and temporal scales likely most relevant at the scale of marine organisms [26]. A principal components analysis (derived from [25]) was used to analyze spatial patterns in SST variability across time, and formed the basis of defining three subregions (Fig 1B, 1C, 1D and 1E). This ensures that areas of coinciding increases and decreases in temperature are not averaged together into a flat time series.

To examine economically important species responses in the model, we incorporated recruitment anomalies from their stock assessment models. We do not have measured recruitment values, so we have to use a model product (stock assessment output). Recruitment anomalies (i.e., the variance in recruitment strength estimated in the model not explained by the size of the spawning stock biomass) were used because they most closely represent overall stock productivity, which we expected would be the most directly measurable impact from climate. Certainly, climate drivers could affect other population processes such as growth or mortality, but estimates of these parameters are not available at the population level. Other available fisheries data such as catch data or abundance indices are not as feasible for use in our analysis; landings are heavily influenced by external management factors, and abundance indices typically contain high levels of observation error and are highly selective toward certain age classes within the population. Finally, it was most logical to expect that the effects of climate drivers on recruitment strength would be immediate (e.g., current temperature and hypoxia conditions would affect the current year of incoming recruits), any lagged effects were thought to be of minimal importance and this simplified the detection of relationships. The net primary productivity (NPP) is a parameter that serves as an index to the biological state of the GoM. Primary production can be altered due to changes in the physical and chemical environment, and thus these dynamics can influence fisheries production [32]. Zooplankton is a fundamental link in the marine food web with a crucial role as both predator and prey to a wide range of trophic levels. Therefore, any changes in the zooplankton community are reflected throughout the marine ecosystem due to this strong linkage [33]. The brown pelican data were the only bird data included because this species occupies higher trophic levels, responds quickly to environmental change and its residency is not restricted to specific areas of the GoM [34]. For additional details on the data and their sources, readers can also refer to [25].

For all ecosystem components, annual averages for the time period 1984–2015 were obtained. Prior to the analysis, the data for each variable were standardized to a mean of 0 and standard deviation 1 to improve the models’ parameter learning by making the means equal and ranges similar.

### Bayesian networks

A BN describes the joint distribution (which is a way of assigning probabilities to every possible outcome over a set of variables, *X*1 …*XN*) by exploiting conditional independence relationships [35]. These relationships are represented by a directed acyclic graph (DAG). The conditional probability distribution associated with each variable *X* encodes the probability of observing its values given the values of its parents, and can be described by a continuous or a discrete distribution. The DAG consists of nodes (or variables) and edges (or links) between the nodes. “Parent” nodes are those from which arrows originate and “child” nodes are those to which arrows are pointing. Edges between nodes represent dependence relationships. Here, the observed variable nodes in the network are Gaussian nodes, so we assume continuous distribution with mean *mu* and covariance *Sigma* [36]. Suppose the continuous-valued node is *Y*, its continuous parents (if any) are *X*, and its discrete () parents (if any) are *Q*. The distribution on *Y* is defined as follows:

Y|X=x,Q=i∼N(mu(:,i)+W(:,:,i)*x,Sigma(:,:,i)) (1)

where N(mu, Sigma) denotes a Normal distribution with mean *mu* and covariance *Sigma* and W is a |Y |*|X|*|Q| regression (weight) matrix [36]. Let |X|, |Y| and |Q| denote the sizes (X and Y are scalars, Q is binary) of X, Y and Q respectively. If there are no discrete parents, |Q| = 1; if there is more than one, then |Q| = a vector of the sizes of each discrete parent. If there are no continuous parents, |X| = 0; if there is more than one, then |X| = the sum of their sizes. Then *mu* is a |Y|*|Q| vector, *Sigma* is a |Y|*|Y|*|Q| positive semi-definite matrix, and W is a |Y|*|X|*|Q| regression (weight) matrix. For more information, please refer to [36]. Each node in the DAG is characterized by a state which can change depending on the state of other nodes and information about those states propagated through the DAG. By using this kind of inference, one can change the state or introduce new data or evidence (change a state or confront the DAG with new data) into the network, apply inference and inspect the posterior distribution (which represents the distributions of the variables given in the observed evidence). Given a graphical structure, BNs naturally perform prediction using inference. Modeling time series is achieved by using an extension of the BN known as the Dynamic Bayesian Network (DBN), where nodes represent variables at particular time slices [37]. DBNs are graphical models of stochastic processes that allow for complex interdependencies between the acting variables [37]. In this study, DBNs allow us to integrate heterogeneous data, specify conditional relationships between those data and make robust predictions of the temporal dynamics of the ecosystem components and their interactions with natural and anthropogenic stressors. DBNs can model the dynamics of a dataset through the use of a hidden variable (HV) that is a variable for which there are missing or unobserved data. When using dynamic state-space models, we can assume that there is an underlying hidden state that generates the observed data and that this hidden state evolves in time as a function of our inputs [37]. A dynamic model represents the behavior of a system over time; however, many systems, including ecosystems, contain non-stationary dynamics. Hidden variables allow us to model these non-stationary system dynamics [38–40]. They are used to represent a change in the interactions of the observed ecosystem components over time. When the model is parameterized with data as in this case, the value of the hidden variable is set to maximize the fit of the model to the data (e.g. the log- likelihood). If the patterns of the observed ecosystem components change in the time series, e.g. the slope of a correlation between two components changes, the value of the hidden variables linked to these components changes. A hidden variable can be linked to one, multiple, or all, of the observed ecosystem components in the model. Then, the hidden variable value depends on all of the observed ecosystem components it is linked to, and a change in the pattern of the hidden variable indicates a change in the system interactions. This is highly useful in ecological analyses where non- stationary dynamics are common and complex ecological interactions change with time due to changing pressures e.g. global climate change [14]; [41].

## Experiments

### Model comparison

**Fig 2:**
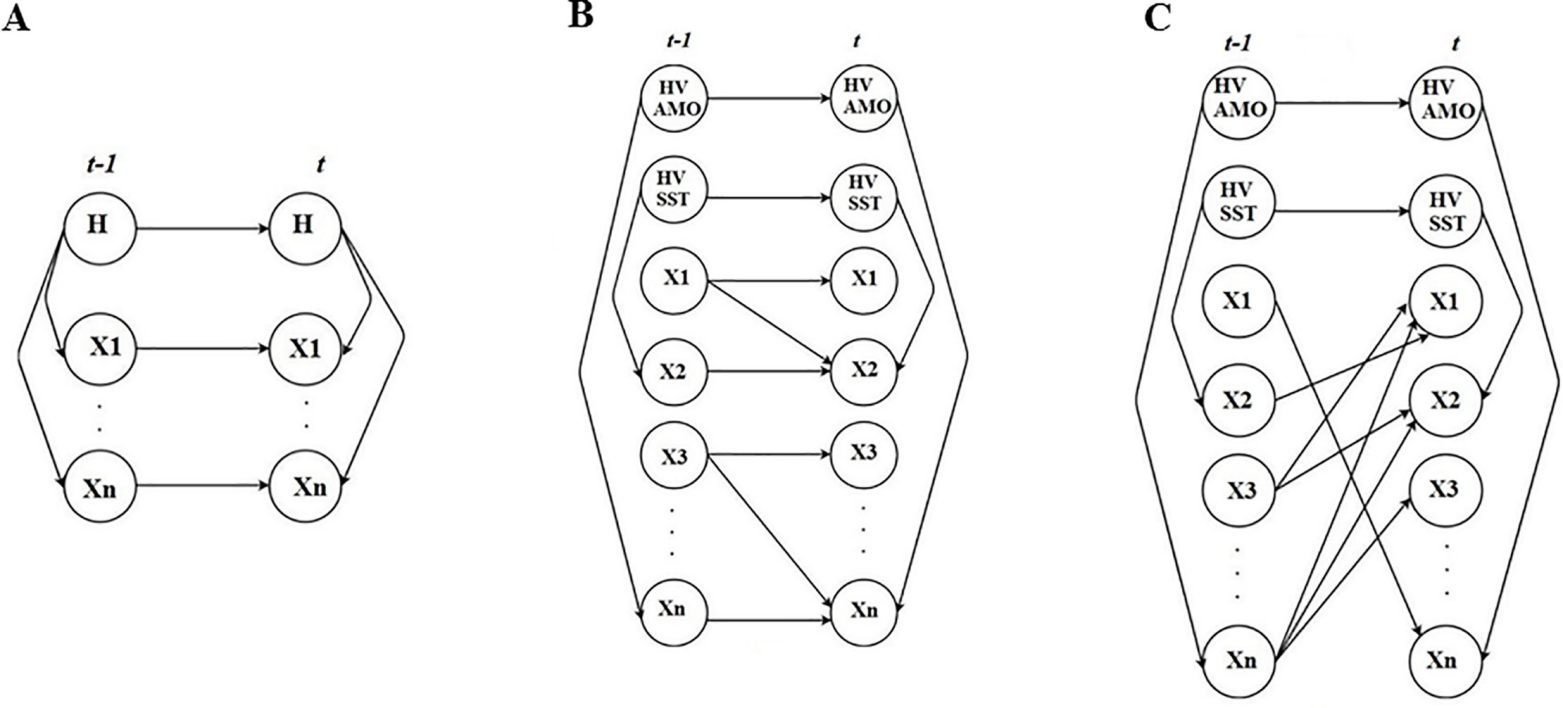
ARHMM, ARDBN and DDDBN models. (A) An autoregressive hidden Markov model (ARHMM), where *H* denotes an unmeasured hidden variable. *X*1 …*XN* denote the measured ecosystem components from Table 1. (B) General structural form of the autoregressive DBN (ARDBN). Note, the autoregressive link is kept and an additional parent node is enforced to different children’s nodes. (C) General structural form of the data-driven DBN (DDDBN). Note, the autoregressive link is removed (except for the two hidden variables) and an additional parent node is enforced with the number of parents varying between the different children’s nodes. These are graphical representations to visualize the differences between the model variants. The actual links between the different *X* nodes are presented in Fig 3. Note, the network linkages and parameters do not change throughout time, the models are time-invariant.

We conduct all experiments using the Bayes Net Toolbox in MATLAB [36]. A series of BN models were built with different levels of structural complexity and uncertainty to model the GoM ecosystem dynamics. Comparing different model structures tests hypotheses regarding the relative role of ecosystem interactions and autoregressive links in the GoM ecosystem. Thus, allowing us to gain an understanding of the ecological complexity and the importance of mechanisms shaping the ecosystem structure. The simplest model was an autoregressive Hidden Markov model (ARHMM). This model assumes a fixed structure and incorporates a single hidden variable which is modeled as a discrete node (Fig 2A). This hidden variable can infer some underlying state of the series when applied through an autoregressive link that can capture relationships of a higher order [37]. The autoregressive structure admits the existence of dependency amongst time series observations while the hidden Markov process could capture the probability characteristics of the transitions amongst the underlying states [37]. Thus meaning, the current observation *X*_*t*_ not only depends on the last observation *X*_*t-1*_ but also on the current hidden state *H*_*t*_.

**Fig 3:**
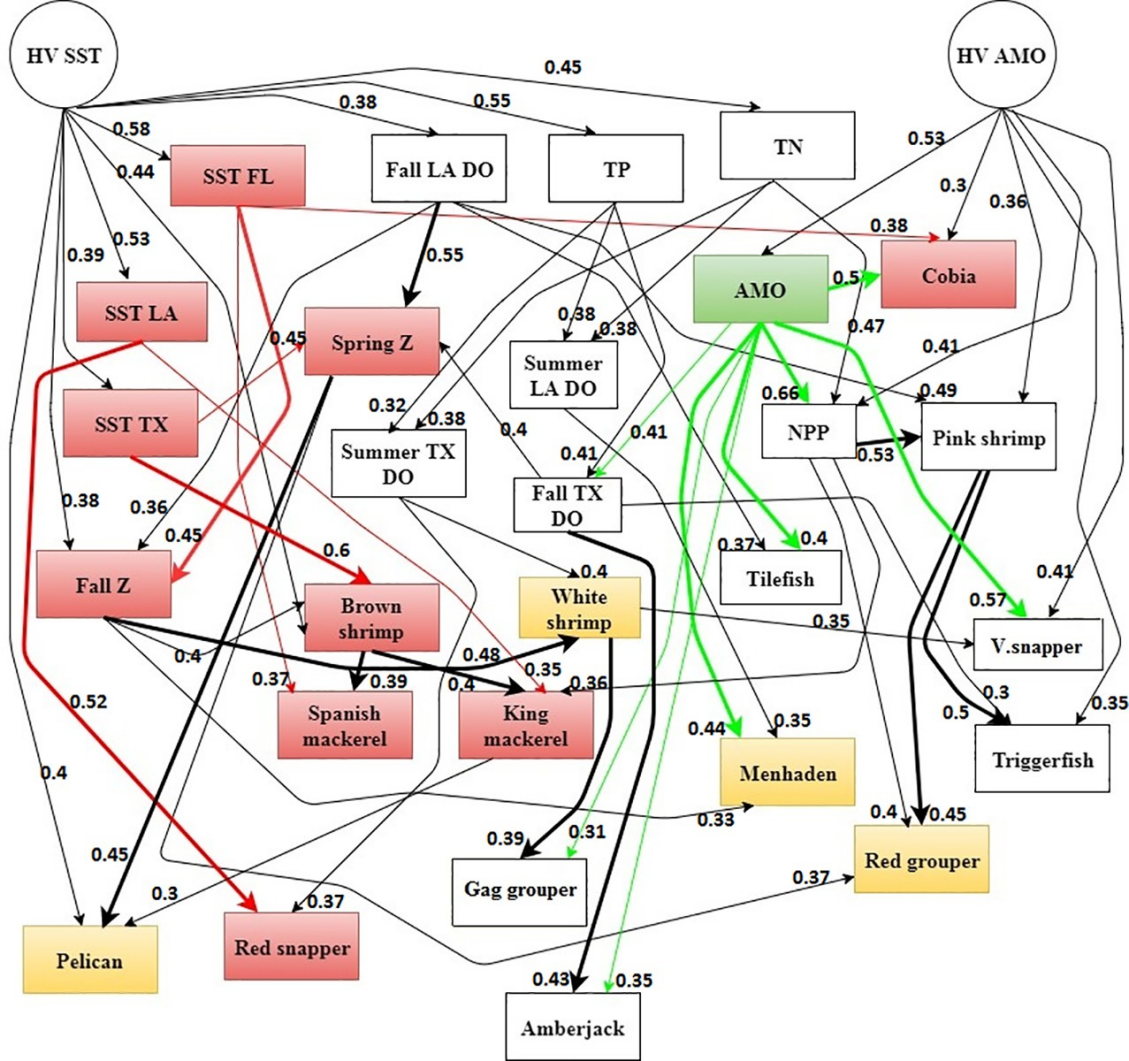
Dependency relationships. The dependency relationships between all the measured ecosystem components and two unmeasured hidden variables (HV AMO and HV SST), learned from the hill- climb, and which were used to construct the DDDBN model. Only the bold links (regardless of color) were used to build the ARDBN. Colors were used to assist visualization of the network. Red colored nodes and arrows denote direct influence by SST. Nodes highlighted in yellow are indirectly influenced by SST. Green colored node and arrows denote direct influence by AMO. The strength of each identified link (i.e. the level of confidence) is also reported.

For the more complex models, a hill-climb technique with random restarts and over a sliding window was applied to learn the dependency relationships among the different ecosystem components and the learned hidden variable from the ARHMM. To minimize the inclusion of insignificant predictors, only those dependency relationships that were of highest confidence, learned from the hill-climb, were included (see Supporting Information for more details on the hill-climb). The learned dependency relationships were used to build the modeling network structures for the parent-child relationships used in the two models described below (Fig 2B and 2C). The moderate complexity model kept the autoregressive link from the ARHMM, utilized two hidden variables and added a single parent node to each child node in the network structure to account for the interactive effect from a single other ecosystem component. This model structure hypothesizes that predictions are optimized by including one additional parameter while still allowing the ecosystem components to be predicted from their previous state. We refer to this model as an autoregressive dynamic BN: ARDBN (Fig 2B). The selection of the additional parameter added to the ARDBN for each ecosystem component was based on the level of confidence from the hill-climb technique (Fig 3). By incorporating two hidden variables into the model, we might be able to define a structure, much closer to the “true” structure of the system we are modelling [37]. By introducing hidden variables into the models, simpler models can be learned that are less prone to over-fitting and more efficient for inference. For example, the apparent complexity of a predicted variable can be explained imagining it as a result of two simple processes, the “true” underlying state, which may evolve deterministically, and our measurement of the state, which is often noisy [37]. We can then “explain away” unexpected outliers in the observations, as opposed to strange fluctuations in “reality”.

One hidden variable (acting as a parent node) was linked to AMO (HV AMO) and another to all the SST indices (HV SST). This was performed to capture any changes in the variance of different ecosystem components and to reflect temporal changes in the underlying environmental processes within the system. The choice of AMO and SST was due to the fact that these climate variables influence regional ecological responses [26] and thus, increase the uncertainty in terms of understanding the effects of climate change against a background of multiple interacting pressures. Note, additional dependency relationships apply between the two hidden variables and the rest of the measured observed ecosystem components in the ARDBN model structure. The learned hidden variable from the ARHMM was incorporated into the data matrix during the hill-climb experiment and any learned relationships were allocated between the two hidden variables in the ARDBN structure. The most complex DBN model was built to include the same two hidden variables from the ARDBN model but removed the autoregressive link and added up to two additional parent nodes for each component, so that the level of complexity in terms of modeling the environmental dynamics was higher and likely more reflective of reality. This model hypothesizes that the “true” generating structure of the ecosystem dynamics relies more upon ecosystem interactions than upon an autoregressive link. The hidden variables, climate and physiochemical drivers, were the only components of the model that maintained an autoregressive link to account for their changes over time. The number of parents to each node varied among the ecosystem components (Fig 2C) but the maximum number of parents was limited to three to avoid over-fitting. This model structure is purely based on data-driven dependency relationships, determined from the hill-climbing procedure, and is thus referred to as the data-driven dynamic BN (DDDBN). By using multiple parent nodes, ecosystem dynamics can be predicted through multiple environmental associations and their changes over time. Moreover, this model can explore predictions of the ecosystem response to changes in climate, while including other interactive effects.

### Generating predictions and modeling hidden variables

Given a graphical structure, BNs naturally perform prediction using inference. The network structure varied with the model frameworks but the method of predicting the variables was universal. Given the probability distribution over **X**[*t*] where **X** = X1 …X*n* are the *n* variables observed along time *t*, to predict the future state of each variable, we inferred the state at time *t* by using the observed evidence (or available data) from *t-1*. Non-parametric bootstrap (re-sampling with replacement from the training set) was applied 250 times for each modeling approach to obtain statistical validation in the predictions and estimates of the standard deviation [35]. Rather than repeatedly obtaining independent data sets from the population, we instead obtain distinct data sets by repeatedly sampling observations from the original data set with replacement. The bootstrapping technique allows obtaining an unknown characteristic of an unspecified distribution by drawing subsets from the observed data iteratively and computing a statistic (standard errors and confidence intervals) for each subset [35]. Bootstrapping lets us obtain approximate distribution of our values and hence to asses bias of our estimate [35]. The data were divided to give the same number of samples for training and a varying number of test pairs. To get the training indices, sampling with replacement (i.e. non- parametric bootstrap) was performed and to get the test indices, those values that are not sampled were used for validation. The process of bootstrapping was performed as follows. First, divide the data to perform training (learning the Bayesian network and applying Expectation Maximization (EM) algorithm [42] for model parameterization), followed by the testing part (model validation). Then, repeat the process for 250 times to be able to identify statistical validation (calculate prediction accuracy) in the model predictions. Model performance, in terms of sum of squared error (SSE), was assessed for each model:

$$\sum {\left(predicted-actual\right)}^{2}$$ (2)

Predictions from the three model variants (ARHMM, ARDBN and DDDBN) were calculated through 2015 and were compared on a year-to-year basis to the measured data. We model the hidden variables based on the values of the observed ecosystem components. We want to compute *P*(*H*^*t*^*|X*^*t*^, *X*^*t−*1^), where *H*^*t*^ represents the hidden variable and *X*^*t*^ *represents* all observed ecosystem components at times *t*. We use the predicted variable states from time *t* to infer the hidden state at time *t*. The hidden variables were parameterized using the EM algorithm in a maximum likelihood sense [42]. In this case, the log-likelihood is:

$$L(\theta )=\log P(X|\theta )=\log\sum_{j}P(X,H|\theta )$$ (3)

where ∑_*H*_ is the sum over the set of hidden variables *H*, required to obtain the marginal probability of the data [43]. Here, the EM algorithm is applied which alternates iteratively between two steps. In the first step of the EM, the hidden variable is inferred using the predicted ecosystem components, whilst in the second step the estimated likelihood function is maximized. When the algorithm converges to a local maximum, the parameters (*θ*) are estimated.

### SST scenarios

The best performing BN, determined from the model comparison, was used to predict how GoM ecosystem components might respond to changes in SST. Specifically, we examined how different ecosystem components respond to increases in SST, accounting for the heterogeneous nature of other driving factors and their changes over time. First, we predict the ecosystem components using historical observations. Then, we designed three SST scenarios to represent the potential impact of climate change. The scenarios investigated were a: 1.0°C, 1.5°C and 3.0°C increase in temperature across all three SST regions. We chose these temperatures to cover a range of potential short and long-term magnitudes of climate change. We manipulate the SST data by increasing it, according to the three scenarios. All other measured ecosystem components remain unchanged. Potential future changes in AMO were not included, because the AMO index is calculated from de-trended SST data [30] and thus it is uncertain how increasing SST may affect AMO. Increasing SST, especially in the summer will likely lower DO by both decreasing the solubility of oxygen and increasing stratification. However, the magnitude of these changes in DO under the three different SST scenarios is uncertain and thus we did not explicitly include changes in DO in the SST scenarios. Thus, the data input for testing each scenario model was the increased SST data alone. The training part of the model learning was unchanged and done on the original measured data and two unmeasured hidden variables. Thus, we keep the historically driven interactions between the ecosystem components and examine their modeled trends under potential changes in SST. The SST scenario models were statistically validated through the same bootstrapping technique described above to obtain a measure of uncertainty.

## Results

### Model comparison

The three model structures: ARHMM, ARDBN and DDDBN displayed variability in their predictive accuracy (Table 2). The majority of the measured ecosystem components (11 out of 18) were best predicted by the DDDBN (see * symbol in Table 2). The remaining components (white shrimp, red grouper, amberjack, Vermillion snapper, tilefish and pelican), were best predicted by the ARDBN and only one variable (red snapper) was most accurately predicted by the ARHMM.

**Table 2:** Sum of squared error (SSE) of ecosystem components predictions generated by ARHMM, ARDBN and DDDBN.

Variable	ARHMM	ARDBN	DDDBN
**NPP**	11.82	11.34	10.74*
**Spring Z**	22.20	21.14	18.70*
**Fall Z**	21.18	22.53	20.17*
**Pink shrimp**	17.62	16.64	15.42*
**Brown shrimp**	20.66	20.92	19.85*
**White shrimp**	24.89	23.02*	25.03
**Menhaden**	31.29	14.32	11.21*
**Cobia**	20.48	19.28	19.05*
**Gag grouper**	13.66	13.02	11.51*
**Red grouper**	23.65	23.16*	24.90
**Red snapper**	16.98*	19.54	19.20
**Spanish mackerel**	21.02	21.91	20.94*
**Greater amberjack**	23.23	18.71*	20.97
**King mackerel**	21.71	17.23	16.13*
**Gray triggerfish**	8.55	5.79	4.83*
**Vermillion snapper**	21.26	16.99*	17.47
**Tilefish**	36.34	22.22*	23.66
**Brown pelican**	22.03	20.06*	23.34

* symbol indicates most accurate predictions among the three models for each individual ecosystem component.

**Fig 4:**
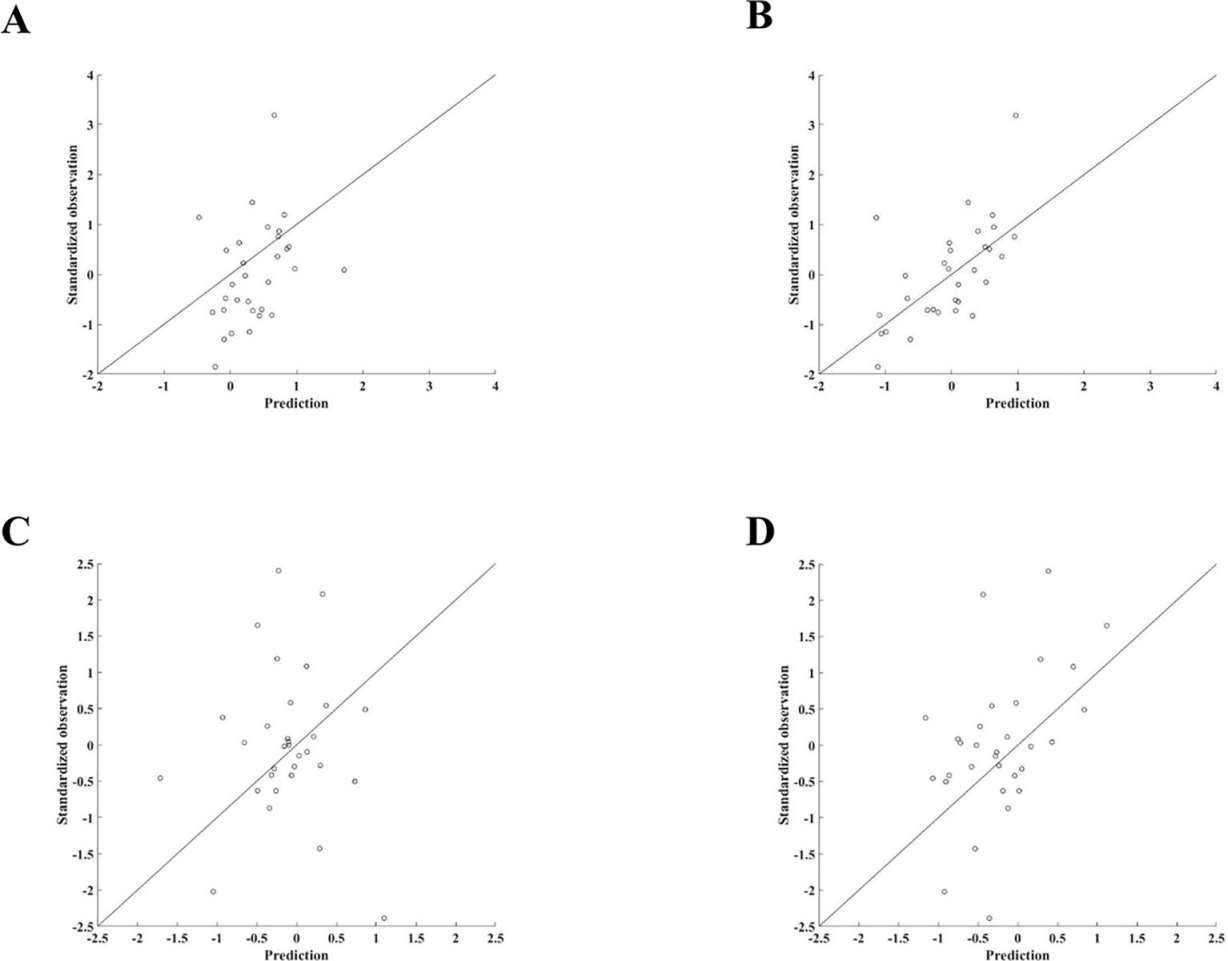
ARDBN and DDDBN model predictions. (A, C) Generated model predictions for the spring and fall zooplankton by the ARDBN and (B, D) DDDBN model respectively. Note the negative scale is due to standardization.

**Fig 5:**
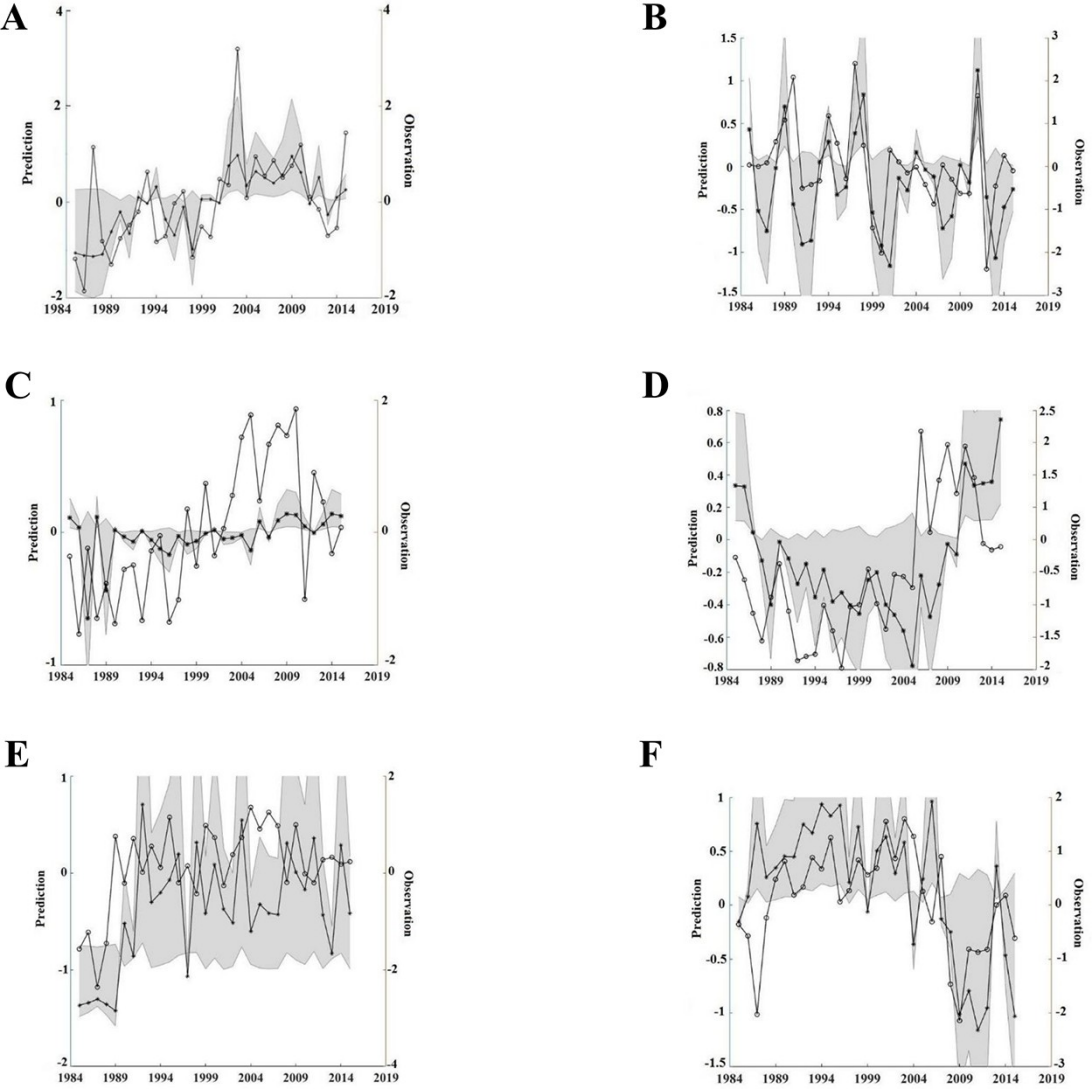
DDDBN model predictions. Generated predictions by the DDDBN model for spring zooplankton (A), fall zooplankton (B), white shrimp (C), brown shrimp (D), red snapper (E) and King mackerel (F). The series marked with stars denote the predictions as opposed to the observed standardized data denoted by circles. 95% confidence intervals (highlighted in gray color) report bootstrap predictions’ mean and standard deviation. Note the negative scale is due to standardization.

In all cases, where the DDDBN did not perform best, it had a similar SSE to the best performing model variant. Interestingly, all of the lower level trophic ecosystem components (except for white shrimp) were most accurately predicted by the DDDBN. There were several upper trophic level fish species (e.g. cobia) that were predicted with relatively equal accuracy across all models. In addition, some ecosystem components were predicted more accurately compared to others across all model types (e.g. gray triggerfish). There were also some relatively high SSEs, e.g. menhaden and tilefish from the ARHMM. We examined the time-series of predicted variable values to determine if they were reproducing the inter-annual variability and long-term patterns observed in the data. We only show some examples but the time-series for all ecosystem components are available in the Supporting information (examples were selected for the main text based on their dependency relationships with SST). Note, we choose to use the two axes plots to best represent the temporal dynamics of the predicted ecosystem components. The DDDBN model had the most accurate predictions for the spring zooplankton, followed by the ARDBN (Fig 4A and 4B). We notice some of the higher magnitude observations, which both models failed to reproduce. Still, the DDDBN model was able to capture the zooplankton dynamics throughout time (Fig 5A), specifically reproducing the positive deviations in the early 2000s and the variations in more recent years, which reflects SST changes during this time period (Fig 1B). To recall, in the model, spring zooplankton is parameterized to be driven by SST off the coast of Texas and dissolved oxygen in the fall in waters off Louisiana and Texas (Fig 3).

For the fall zooplankton, the DDDBN model projected most accurate predictions, followed by the ARDBN (Fig 4C and 4D). Similarly, both models failed to capture some of the outliers, although we can see that the DDDBN model reproduced the temporal dynamics for this ecosystem component (Fig 5B). Specifically, the model reflected well the variations until the late 1990s and the decrease in early 2000s. Predictions from the DDDBN were sensitive to the input data, but overall the model was able to capture temporal trends of the different ecosystem components, e.g. increase in brown shrimp (Fig 5D) and decline in King mackerel (Fig 5F). The model was able to recreate the dynamics of the ecosystem components in time but in some cases it failed to reproduce individual year effects (e.g. year 1987 for spring zooplankton and King mackerel), which could be due to some noise in the data, sampling variation or a mechanism not included in the model. The red snapper was characterized with some variability throughout time (Fig 5E), but the model was able to recreate the temporal dynamics, except for some individual years. Similarly, the white shrimp was characterized with some variation throughout time but the model was able to capture the increase in early 2000s (Fig 5C).

### SST scenarios

**Fig 6:**
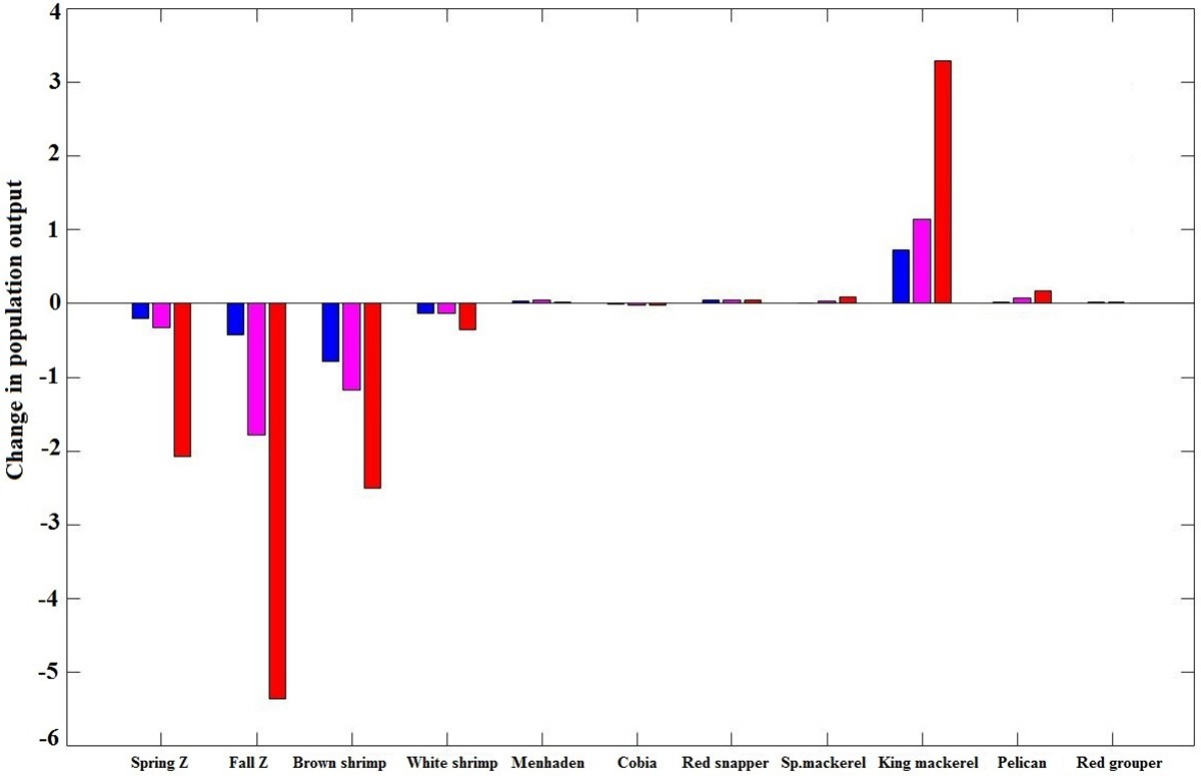
Change in population output. Mean difference of change between the estimated population outputs of the DDDBN model and SST scenarios: increase of 1.0°C (blue), 1.5°C (purple) and 3.0°C (red).

We increased SST for input into the DDDBN model to examine potential GoM ecosystem responses to increasing temperature scenarios (1.0°C, 1.5°C and 3.0°C). We compared the projected outputs among the SST scenarios by examining the response of different ecosystem components. We explored the effect of temperature increase on ecosystem components that are either directly influenced by SST or indirectly influenced by SST through a single intermediary component, according to the network structure, shown in Fig 3 (that is 11 out of the total 18 ecosystem components, which are shown in Fig 6). The results show some variability in the projected response of the examined ecosystem components to increasing SST. The most pronounced effect was a decrease in all lower trophic level ecosystem components, especially zooplankton and brown shrimp (Fig 6). The effect was not as pronounced and more variable in direction for the higher trophic level fish species. The largest effect in upper trophic levels was on King mackerel.

**Fig 7:**
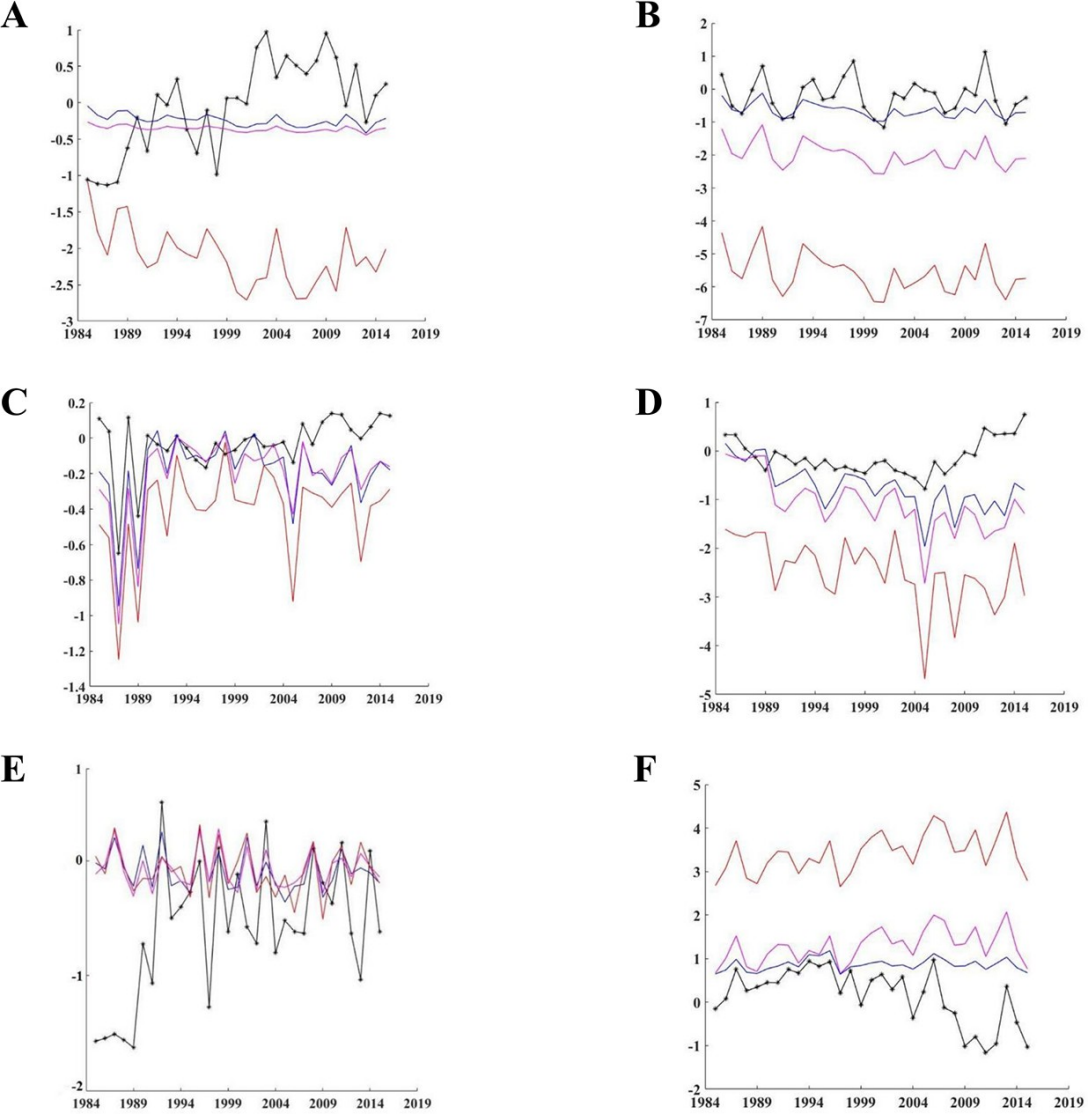
SST scenarios. Generated predictions by the DDDBN model (star symbol) versus the 1.0°C (blue line), 1.5°C (purple line) and 3.0°C (red line) SST scenarios. Note, the negative scale is due to standardization.

We show the DDDBN model and scenario model outputs throughout time for selected ecosystem components from Fig 6, based on the magnitude of response. The spring zooplankton biovolume, following the 1.0°C and 1.5°C increased SST scenarios, was predicted to be relatively stable prior to 2000 and lower than the DDDBN model after 2000 (Fig 7A). A much stronger decline with increased variability was modeled throughout the study period under the 3.0°C increased SST scenario. Contrarily, fall zooplankton biovolume was reduced for the entire study period under all increased temperature scenarios, but inter-annual variability was similar to the DDDBN model. The decline was more pronounced and variability increased for the scenarios with larger SST increases (Fig 7B). Similarly, white (Fig 7C) and brown shrimp (Fig 7D) populations decreased under all increased SST scenarios with greater decreases predicted in the scenarios with greater increases in temperature. Brown shrimp decreased more than white shrimp in all SST scenarios. This might be the result of a direct SST effect on the brown shrimp but intermediary SST effect on the white shrimp through changes in the fall zooplankton dynamics. All SST scenario outputs appeared to show similar inter-annual variability and patterns to one another for both white and brown shrimp, but all showed increased variability compared to the baseline output and the variability increased with increasing SST.

There was not a significant change on the red snapper dynamics (Fig 7E), following the increased SST scenarios and the inter-annual variability remained constant. King mackerel increased under all SST scenarios with the increases greater as SST increased and the highest variability under the larger SST increase but a similar inter-annual pattern in all three scenarios (Fig 7F).

## Discussion

The different model structures considered in this study allow for a comparison of the importance of internal ecosystem component dynamics versus external drivers or connections between components, and allow for an assessment of how model complexity affects model performance. The ARHMM is a relatively simple modeling approach that undertakes a fixed structure, which is not able to account for any ecosystem interactions and predicts the component based only on its prior value and relationship to a hidden variable (Fig 2A). The ARHMM was the worst performing structure and was only best able to predict a single ecosystem component; even for that component, its improvement was slight and unlikely significant compared to the other two model structures (Table 2). The ARDBN performed better than the ARHMM due to the inclusion of a single ecosystem interaction on the predicted ecosystem component. There were some similarities in the performance between the ARHMM and ARDBN, which is likely due to the autoregressive link being present in both models. The ARDBN outperformed the DDDBN for some ecosystem components, although, these improvements over the DDDBN were usually slight. The dynamics of ecosystem components better predicted by the ARDBN suggest a dependence on their prior condition. Alternatively, other factors, such as habitat availability, which we did not include in the model, might be driving their temporal variations to a greater extent than other ecosystem effects. The improved performance in the ARDBN model for some of the ecosystem components (most notably tilefish, greater amberjack and Vermillion snapper recruitment) suggests higher influence of the prior status and a single ecosystem interaction (e.g. AMO for tilefish and Vermillion snapper, and dissolved oxygen for greater amberjack; Fig 3) compared to multiple ecosystem interactions for these ecosystem components. The best performing model, the DDDBN, accounted for up to three ecosystem interactions, two hidden variables and their changes over time, but it did not have an autoregressive link. This suggests the need to include ecosystem effects and distributional heterogeneity, when building predictive models of diverse and heavily exploited ecosystems, such as the Gulf of Mexico. One of the biggest differences between this dynamic Bayesian network modeling approach and other more traditional approaches is the incorporation of two hidden variables. Hidden variables capture changes in ecosystem variance that might not be represented within the model structure. Indeed, the accurate performance of the DDDBN and ARDBN models was likely due, at least in part, to the inclusion of the two hidden variables that reduced the likelihood of introducing spurious interactions into the analysis and allowed for more plausible network structures. The comparative evaluation of different structures, in terms of modeling the Gulf dynamics, showed that predictive performance of the ecosystem components was improved when multiple interactions within their environment were included, compared to prediction based more upon the previous state of the ecosystem com- ponent.Specifically, the DDDBN model suggests the importance of accounting for the effects of temperature and AMO on the dynamics of higher trophic level species. Also, the importance of factors like dissolved oxygen concentration on some of the economically important species (e.g. menhaden) suggests the environment plays a significant role in fishery dynamics; perhaps an even greater role than management actions for some fishery species [44]. For example, gray triggerfish has almost consistently decreased in abundance over the past decades, despite implementing a rebuilding plan with regulations to reduce fishing pressure [25]. In the DDDBN model, gray triggerfish recruitment is influenced by the dynamics of the HV AMO, pink shrimp and oxygen concentration, the latter two having a generally decreasing trend in recent years (S2 Fig). The combination of these physical and biological associations might be controlling, to some extent, the population dynamics of gray triggerfish. Most importantly, our study highlights AMO as a proxy for a number of complex processes that simultaneously affect the biology of the Gulf ecosystem. Also, our results emphasize the importance of hypoxia in terms of influencing fisheries productivity and trophic structure of the system [26]; [45]. Similarly, it has been shown for other systems that more complex models result in better predictive performance [46]. For the North Sea, similar data-driven techniques have been applied to model fish biomass and a more complex model-ing structure was found to perform better in terms of predicting species dynamics across space and time [14]. In general, studies have suggested that non-linear modeling approaches (i.e. classification trees and neural networks) are better able to capture and model complex patterns, found in ecological data [47]. The DDDBN model represents a flexible framework of medium complexity between single-stock assessments and multi-species mechanistic models, such as Ecopath with Ecosim and Atlantis. Moreover, the DDDBN model struc-ture does not rely upon an assumption of steady state, which is unlikely to be met in ecosystems. This allows the model structure to use changes in temporal distributions and structure to inform the model and its predictions. By extending our DDDBN to model temperature scenarios, we allow for probable climate change impacts to be predicted. Ecosystem components varied in their sensitivity and directional response to increasing temperature. The variability to changes in temperature is potentially the result of ecosystem components-specific effects and interactions within their environment. In addition, differences in the predicted temperature responses could be explained by the specific thermal tolerance of the ecosystem component, its prey, its predators, or the multiple interacting stressors within its habitat. Despite this variability, we see some universal response across trophic levels that could provide insights about ecological stability and resilience in a changing climate. Changes in temperature had large impacts on zooplankton and shrimp dynamics and all reduced their values with increasing temperature. Spring zooplankton biovolume showed less prominent decrease with increasing temperature over the 30- year study period; whereas, fall zooplankton biovolume was systematically lower under all increased temperature scenarios with the higher increases in temperature predicting greater decreases in fall zooplankton biovolume. The projected dynamics of the spring zooplankton (calculated from the open ocean survey) are influenced by temperature changes but perhaps even more strongly controlled by the AMO current warm phase (Fig 1A), which potentially led to the projected sta-bility in the scenario trends compared to the DDDBN output (Fig 7A). The AMO has been invoked as the explanatory factor for a number of biological phenomena in the Atlantic Ocean and GoM [29]; [48]. In addition, AMO has some indirect influence on the extent and magnitude of hypoxia, thus impacting concentrations of zooplankton in the nearshore waters [26]. The fall zooplankton survey covers stations located on the continental shelf, where it has been shown that oxygen concentrations can affect the structure of biological communities, having positive effects on some living marine resources and negative on others [49]. There is some evidence about the tolerance of low oxygen concentrations between the co-dominant zooplankton species in the GoM: *Acartia* spp. and *Centropages* spp., with the latter being more abundant in low oxygen waters [49]. Variability of the zooplankton community with environmental conditions has implications for the quality of the food environment for larval and planktivorous fish [49]. White shrimp and brown shrimp recruitment had consistent reductions for all temperature scenarios with reductions larger when temperatures were increased more. Brown shrimp reductions were more pronounced, compared to the white shrimp, for all temperature scenarios (Fig 6). Laboratory studies have suggested that brown shrimp might be more sensitive to temperature changes and survival of juvenile shrimp within coastal waters of Texas (here, brown shrimp was influenced by Texas SST) decreases with high temperatures, supporting the dramatic decreases predicted in our model for brown shrimp [50–51]. The white shrimp showed similar responses to 1.0°C and 1.5°C temperature increases, but larger decreases in the 3.0°C increase SST scenario. In the model, white shrimp was not directly influenced by temperature, but was driven by changes in fall zooplankton biovolume that is influenced by Florida shelf SST. The brown and white shrimp results are consistent with the observed temperature changes within the different shelf areas (Texas and Florida SST) and their trends in the last 5 years (Fig 1, increasing for Texas and more or less stable for Florida). These results suggest that ecosystem responses to any future changes in temperature will be influenced by the spatial habitat. Spatial variations in temperature potentially lead to spatial variability in productivity, which subsequently causes further forcing on higher level trophic species and mixture of responses at spatial scales. These decreases in shrimp and zooplankton will have implications for the availability of the food environment for upper trophic level species, including finfish of high economic importance [49]. The response to increasing SST was more variable and less intense for upper trophic levels; although, King mackerel showed the largest increase of any ecosystem component in all three temperature scenarios (Fig 6). This response might be a result of habitat preferences of the King mackerel and its high temperature tolerance [52]. A study of mackerel landings reported higher catches with increasing warmer temperatures, specifically during La Nina winters [53]. In addition, the mackerel was driven by the dynamics of NPP, which was predicted to be relatively stable with some increase from early 2000 (S1 Fig), potentially explaining the positive response in this fish species. For some higher level trophic species, such as cobia, we were not able to see any definitive changes in response to increased SST. Cobia dynamics were influenced by AMO, so similarly, such results could be explained with the current AMO warm phase, masking the full effect from temperature change. It has been shown that AMO has influenced North Atlantic fisheries since the early 1900s [29] and is correlated with community- wide fishery responses in seven northwestern Atlantic ecosystems [28]. Similarly, we were not able to detect significant impact on the red snapper, following increases in temperature. According to [54], changes in coastal wetland habitats due to sea level rise and changes in rainfall and freshwater flow patterns may be among the most important drivers of climate change impact on species like the red snapper. Generally, red snapper are widely distributed although they have a high affinity for certain habitat types at various stages in their development [55]; thus, we would not necessarily expect their population dynamics to be specifically sensitive to temperature. In our model, the red snapper was influenced by the dynamics of dissolved oxygen, which might play a more significant role for this species population dynamics, compared to SST. These results suggest that some ecosystem components will be negatively impacted, others such as the King mackerel may benefit from the effects of climate change or may be more resilient to changes in their environment such as the red snapper, showing little impact from increasing temperatures. This finding is consistent with other ecological predictions of climate change effects on marine ecosystems, using a variety of methods [56–58]. Our study highlights that to assess potential “winners and losers” in a changing environment, factors like trophic associations and interactions with physical factors, affected by climate change, must be taken into consideration to evaluate the population dynamics and processes in a comprehensive manner. In addition, our study is a useful, independent comparison to mechanistic approaches designed to derive the same predictions in the GoM, such as the Climate Vulnerability Analysis [56]. There were some high SSEs (Table 2) and some outliers from the observed data (Fig 4), that the DDDBN model did not capture well. In addition, there was some similarity in accuracy from the different models that might be attributed to the similar effects of changing climate on many species [58]. In particular, stock assessment estimates are subject to multiple sources of uncertainty, which can be categorized into three types: observation, structural and estimation, according to [59]. In some cases, process error is considered to be a special case of structural uncertainty, however that is being accounted for here by using state-space models that simultaneously estimate the variances, associated with observation and process errors, using maximum likelihood [60]. Estimation uncertainty can be characterized by calculating Hessian- based confidence intervals or as we have done here by bootstrapping or in a Bayesian context as probability intervals from the posterior distribution of estimates. Our comparative evaluation of multiple model structures that reflect different hypotheses of the GoM ecosystem complexity is one way of dealing with structural uncertainty. Of particular importance is the use of multiple lines of evidence to support conclusions, considering alternative hypothesis, and accounting for uncertainty [59]. The data preparation and analysis, specifically standardization and non-parametric bootstrap, increase confidence in these results and the modeling structure applied. Most importantly, we account for the observation uncertainty by introducing hidden variables into the models, which allow for simpler models to be learned that are less prone to over- fitting and more efficient for inference. In most domains, the observed variables represent only some characteristics of a system, which can have a negative effect on the learning procedure. In this work, the hidden variable was chosen to most easily reflect the complex interdependencies between and among ecosystem components and their environment and account for any spurious relationships that might degrade the precision and accuracy of the results. One aspect of the underlying model structure that could be further improved would be to include socio-economic variables, for example commercial fishery landings, that would allow the model to provide information to managers that includes predicting socioeconomic impacts. The model could also be expanded to include other ecosystem components, such as protected species and microbial data to address issues like biodiversity and survival and support management strategies for protected species population recovery. In addition, the model could be developed on a more localized scale to investigate impacts from hypoxia and eutrophication. Marine populations are being threatened by both natural and anthropogenic sources and in order to understand issues between sustainability and management, populations cannot be addressed in isolation, but in relation to their interactions and associations with external factors. Data-driven techniques allowed us to strengthen our knowledge on the mechanisms involved in shaping the functional ecological network within the GoM, making them less prone to error, by not introducing assumptions about a complex system. The DDDBN modeling approach that accounted for multiple ecosystem interactions and their changes over time further highlighted the importance of distributional heterogeneity and ecosystem components-specific effects, when building predictive models of such diverse and exploited ecosystems. We explored potential ecosystem changes in response to increasing temperature in the GoM by applying temperature scenarios to the DDDBN model. The applied methods here have extended our knowledge into the complexity of the region and its ecological structure and resilience that will potentially help addressing applied questions in the field of fisheries management. Future work will involve extending the applied network model further into the future to explore the effect of alternative management strategies on specific economically important species and using summer versus winter SST values to address seasonal differences in spawning. In addition, projections from climate models (e.g. [60]) could be used in combination with the DDDBN model to investigate downscaling outputs.

## Supporting information

S1 Supporting Information Hill-climb description and additional Figures. (PDF)

S1 Table Model bias. (TIF)

S1 Fig DDDBN model predictions. Generated predictions by the DDDBN model. The series marked with stars denote the predictions as opposed to the observed data denoted by circles. 95% confidence intervals report bootstrap prediction’s mean and standard deviation.
(TIF)

S2 Fig Pink shrimp and oxygen concentration. (A) Pink shrimp recruitment deviation. (B) Bottom water dissolved oxygen concentration for the Texas coastal shelf in fall.
(TIF)

S1 File Data. Data used in this study.
(XLSX)
