# Supplementary material for: Predicting ecosystem components in the Gulf of Mexico and their responses to climate variability with a dynamic Bayesian network model
Source: PLoS One. 2019 Jan 23;14(1):e0209257. doi: 10.1371/journal.pone.0209257 (PMC6344104; doi:10.1371/journal.pone.0209257)
Supplement: S1 Supporting Information — (PDF) [file pone.0209257.s001.pdf]

S1 Supporting Information. Hill-climb Description and Additional Figures.

December 11, 2018

1 Hill-Climb

The hill-climb is an optimization technique that belongs to the family of local search. The search begins with an empty network. In each stage of the search, networks in the current neighborhood are found by applying a single change to a link in the current network such as *add arc* or *delete arc* and choose the one change that improves the score the most. We performed the hill-climb with random restart (number of random restarts = 10) which conducts several hill-climbing runs, perturbing the result of each one as the initial network for the next [1]. The learned network with the highest score is kept and if a new run of hill climbing produces a better network than the stored one, it replaces the stored one. Random restarts were utilized to measure the confidence of each interaction being in the network, not just examine the dependency relationships. The learned BN links represent dependence, these are relationships that are predictive in an informative, not causal manner. We used the Bayesian Information Criterion (BIC, [2]) for scoring candidate networks:

$$BIC = logP(D|theta\_hat) - 0.5^{*}d^{*}log(N), \quad (1)$$

where D is the data, theta_hat is the maximum likelihood estimate of the parameters, d is the number of parameters, and N is the number of data cases.

Score-based approaches (such as the hill-climb) consist of forming a set of possible network structures, each represented by a score of how well it fits the data. Then, the network structure with the highest score is selected. The hill-climb does not perform in two stages. The hill-climb (with random restarts) is conducted simultaneously. At each iteration, the BIC is computed to determine if the new network fits the data better than the previous one. The algorithm stops when there is no more improvement and the final network is selected.

In addition, to learn the network structure for each year in the time window, the hill-climbing was conducted on a sliding window of data (size of window = 10 years). In this way, we would be able to capture any significant functional interactions over the previous 10 years. Then, the hill-climb learning was performed for 1500 iterations. We define a confidence threshold- the minimum

confidence (estimate of the probability of finding a link) for an edge (or a link) to be accepted as an edge in the learned network structure, following convergence of the hill-climb. We defined interactions of high confidence in time as those in which we have the greatest mean confidence (≥ 0.3) of being in the generated network.

We defined interactions of high confidence in time as those for which we have the greatest mean confidence of being in the generated network. The choice of the 0.3 threshold was based on the range (the maximum was 0.66 and the minimum was 0) of mean confidence found from the hill-climb. Based on the range of values, a threshold of 0.2 was thought to be too low and a threshold of above 0.3 to be too high. Simple deduction experiments were conducted to explore the sensitivity of the threshold choice and it was adequately found that the threshold of 0.3 is a sufficient choice. Future work could look into the remaining discovered dependencies (below the 0.3 threshold) to understand the reasoning for their confidence.

Assessing convergence of the hill-climb technique was performed based on varying the number of iterations and examining the learning curve for the BIC score. The choice of 10 random restarts was selected based on the complexity of the model and the length of the data series. [3] showed that a few random restarts suffice when the model applied is relatively small and variables are strongly dependent on each other. In the case of using data, structure learning is a *NP*-hard problem because the number of directed acyclic graphs (DAGs) on N variables is super-exponential in N, thus we cannot exhaustively search the space. It is not possible to give a simple answer to how many restarts should be used because it depends on the model [4]. It is indeed recommended in such cases to use methods to look for local optima in the structure space, e.g. score based learning algorithms, for example hill-climbing. The choice of 10 random restarts has been considered adequate in the present study, considering the purpose and ecological significance of the study. The technique of utilizing hill-climb with random restarts has been proven useful in the case of ecological analysis for other ecosystems (North Sea: [5] and Gulf of St Lawrence: [6]), where data sets of similar size and modeling techniques have been used.

We report the identified strength (from the hill-climb) of each link:

HV SST ⟶ TN (0.45), TP (0.55), Fall LA DO (0.38), SST FL (0.58), Brown shrimp (0.44), SST LA (0.53), SST TX (0.39), Fall Z (0.38), Pelican (0.4)

HV AMO ⟶ AMO (0.53), Cobia (0.3), Pink shrimp (0.36), NPP (0.41), Triggerfish (0.35) and Vermillion snapper (0.41)

SST FL ⟶ Cobia (0.38), Fall Z (0.45), Spanish mackerel (0.37)

Fall LA DO ⟶ Fall Z (0.36), Spring Z (0.55), Tilefish (0.37), Pink shrimp (0.49)

TP ⟶ Summer TX DO (0.32), Summer LA DO (0.38), Fall TX DO (0.41)

TN ⟶ Summer TX DO (0.38), Summer LA DO (0.38), NPP (0.47)

SST LA ⟶ Red snapper (0.52), King mackerel (0.35)

Spring Z ⟶ Pelican (0.45), Red grouper (0.37)

Summer LA DO ⟶ Menhaden (0.35)

AMO ⟶ Fall TX DO (0.41), Menhaden (0.44), Gag grouper (0.31), Amberjack (0.35), Tilefish (0.4), NPP (0.66), Vermillion snapper (0.57), Cobia (0.5)

SST TX ⟶ Brown shrimp (0.6) and Spring Z (0.45)

Summer TX DO ⟶ Red snapper (0.37), White shrimp (0.4)

Fall TX DO ⟶ Amberjack (0.43), Spring Z (0.4) and Triggerfish (0.3)

NPP ⟶ Red grouper (0.4), Pink shrimp (0.53) and King mackerel (0.36)

Pink shrimp ⟶ Red grouper (0.45), Triggerfish (0.5)

Fall Z ⟶ Brown shrimp (0.4), White shrimp (0.48), Menhaden (0.33)

Brown shrimp ⟶ Spanish mackerel (0.39), King mackerel (0.4)

White shrimp ⟶ Gag grouper (0.39), Vermillion snapper (0.35)

King mackerel ⟶ Pelican (0.3)

S1 Table. Model bias.

Variable	ARHMM	ARDBN	DDDBN
**NPP**	0	0.05	-0.13
**Spring Z**	0.04	-0.09	-0.06
**Fall Z**	0.05	-0.15	-0.12
**Pink shrimp**	0.05	-0.11	0.03
**Brown shrimp**	0.02	-0.09	-0.17
**White shrimp**	0.005	0.14	-0.02
**Menhaden**	0.005	-0.09	-0.15
**Cobia**	-0.09	-0.14	-0.005
**Gag grouper**	-0.0019	0.15	0.14
**Red grouper**	-0.11	-0.06	0.17
**Red snapper**	0.05	0.12	0.15
**Spanish mackerel**	-0.01	0.09	0.12
**Greater amberjack**	-0.08	-0.02	-0.14
**King mackerel**	0.04	-0.05	-0.12
**Grey triggerfish**	0.05	-0.01	0.01
**Vermillion snapper**	-0.01	-0.15	-0.07
**Tilefish**	0.14	0.13	0.04
**Brown pelican**	0.12	-0.15	0.08

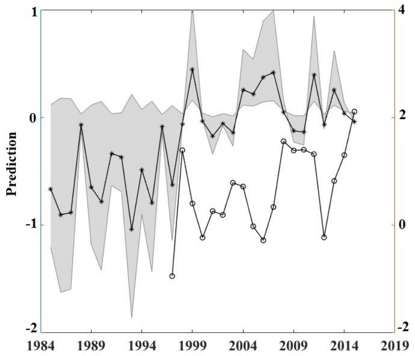

(a) NPP, DDDBN model

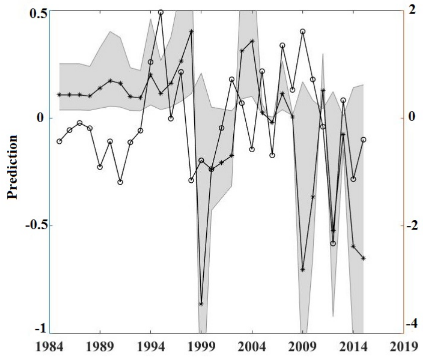

(b) Pink shrimp, DDDBN model

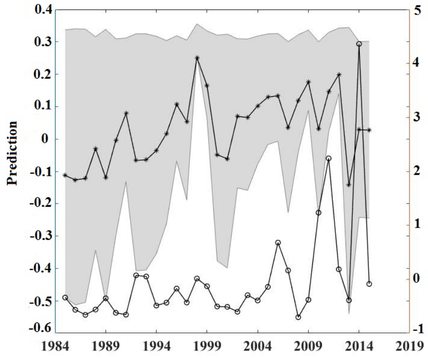

(c) Menhaden, DDDBN model

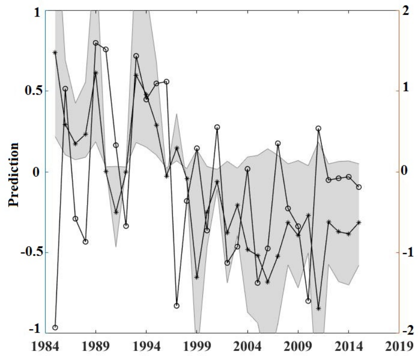

(d) Cobia, DDDBN model

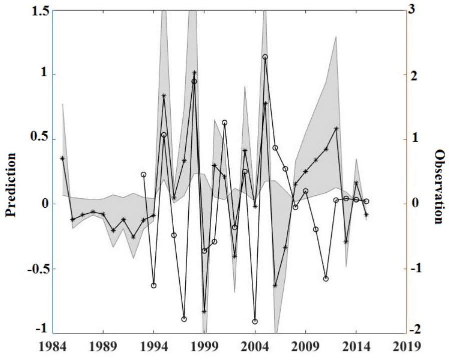

(e) Red grouper, DDDBN model

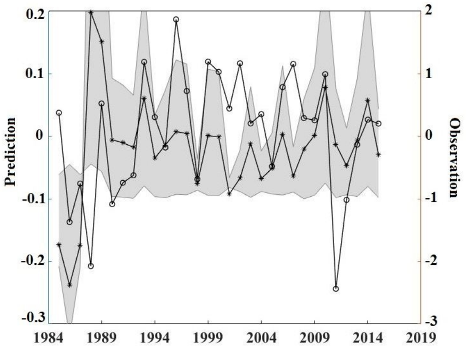

(f) Gag grouper, DDDBN model

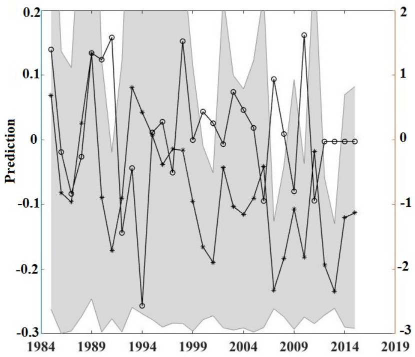

(g) Spanish mackerel, DDDBN model

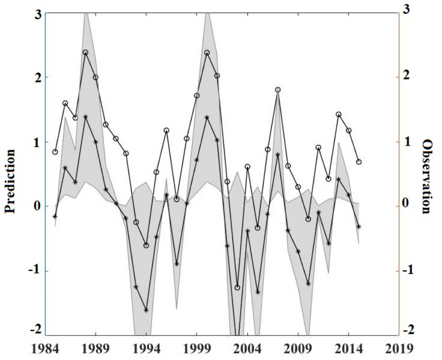

(h) Greater amberjack, DDDBN model

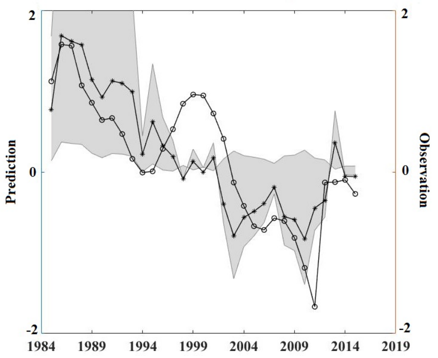

(i) Grey triggerfish, DDDBN model

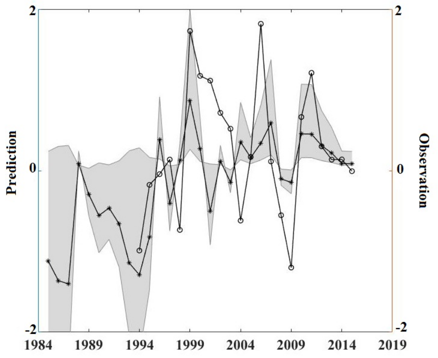

(j) Vermillion snapper, DDDBN model

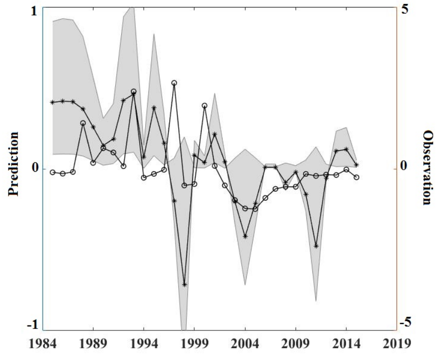

(k) Tilefish, DDDBN model

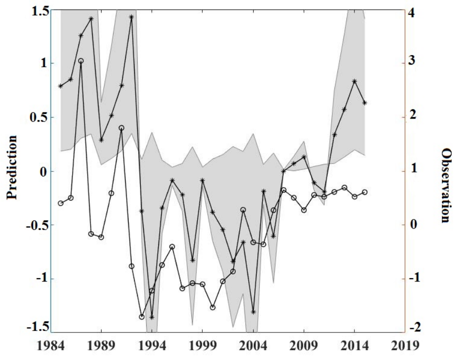

(l) Brown pelican, DDDBN model

S1 Fig. Generated predictions by the DDDBN model. The series marked with stars denote the predictions as opposed to the observed data denoted by circles. 95% confidence intervals report bootstrap predictions' mean and standard deviation.

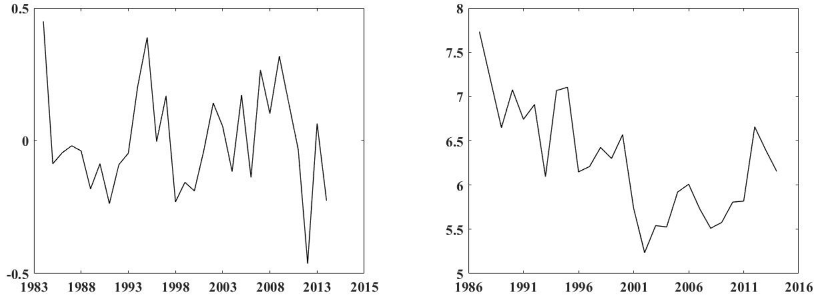

(a) Pink shrimp (b) Oxygen concentration

S2 Fig. (A) Pink shrimp recruitment deviation. (B) Bottom water dissolved oxygen concentration for the Texas coastal shelf in fall.

References:

[1] Bouckaert R. Bayesian belief networks: from construction to inference. PhD diss., 1995.

[2] Murphy K, et al. The Bays net toolbox for Matlab. Computing science and statistics. 2001; 33(2): 1024- 1034.

[3] Wang Y and Zhang L. Severity of local maxima for the EM algorithm: Experiences with hierarchical latent class models. In Proceedings of the Third European Workshop on Probabilistic Graphical Models (PGM-2006).

[4] Mourad R, Sinoquet C, Zhang N, Liu T, Leray P. A survey on latent tree models and applications. Journal of Artificial Intelligence Research. 2013; 47: 157- 203.

[5] Trifonova N, Maxwell D, Pinnegar J, Kenny A and Tucker A. Predicting ecosystem responses to changes in fisheries catch, temperature, and primary productivity with a dynamic Bayesian network model. ICES Journal of Marine Science. 2017; 74(5): 1334- 1343.

[6] Trifonova N, Duplisea D, Kenny A, Tucker A. A spatio-temporal Bayesian network approach for revealing functional ecological networks in fisheries. In International Symposium on Intelligent Data Analysis. 2014; 298-308. Springer, Cham.
