# Supplementary figures and images for: Predicting ecosystem components in the Gulf of Mexico and their responses to climate variability with a dynamic Bayesian network model
Source: PLoS One. 2019 Jan 23;14(1):e0209257. doi: 10.1371/journal.pone.0209257 (PMC6344104; doi:10.1371/journal.pone.0209257)

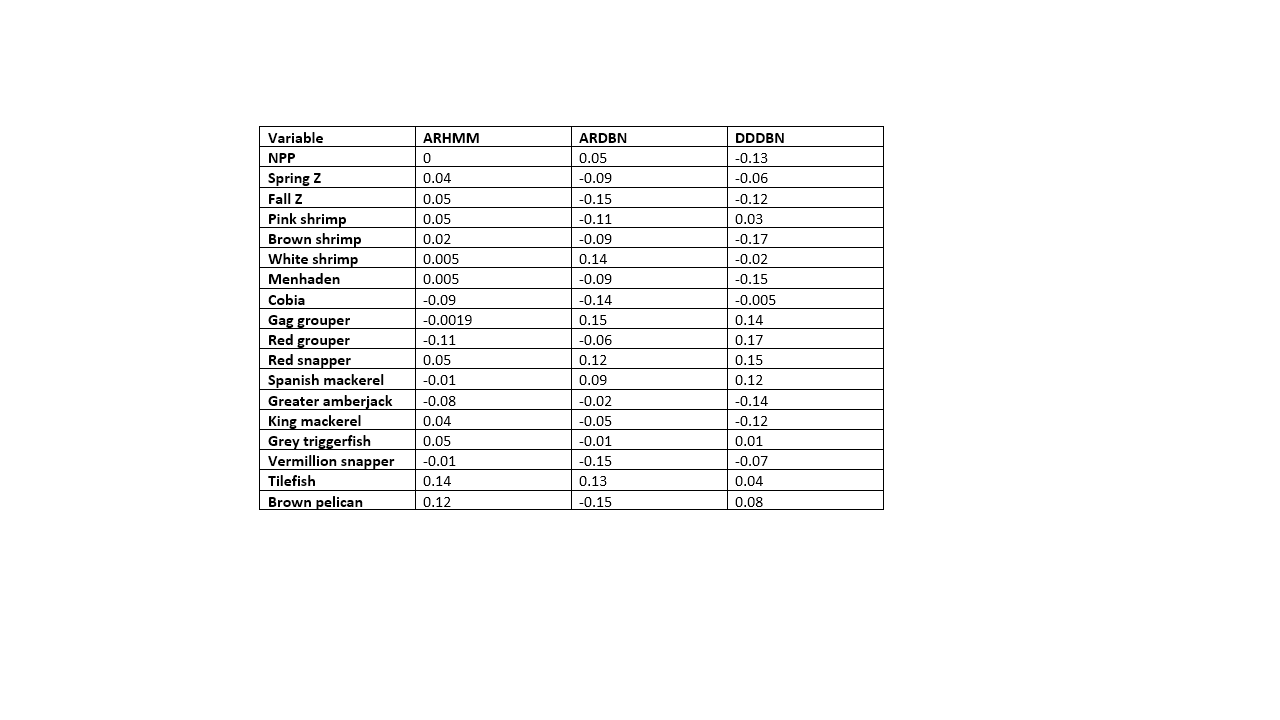

Supplement: S1 Table — (TIF) [file pone.0209257.s002.tif]

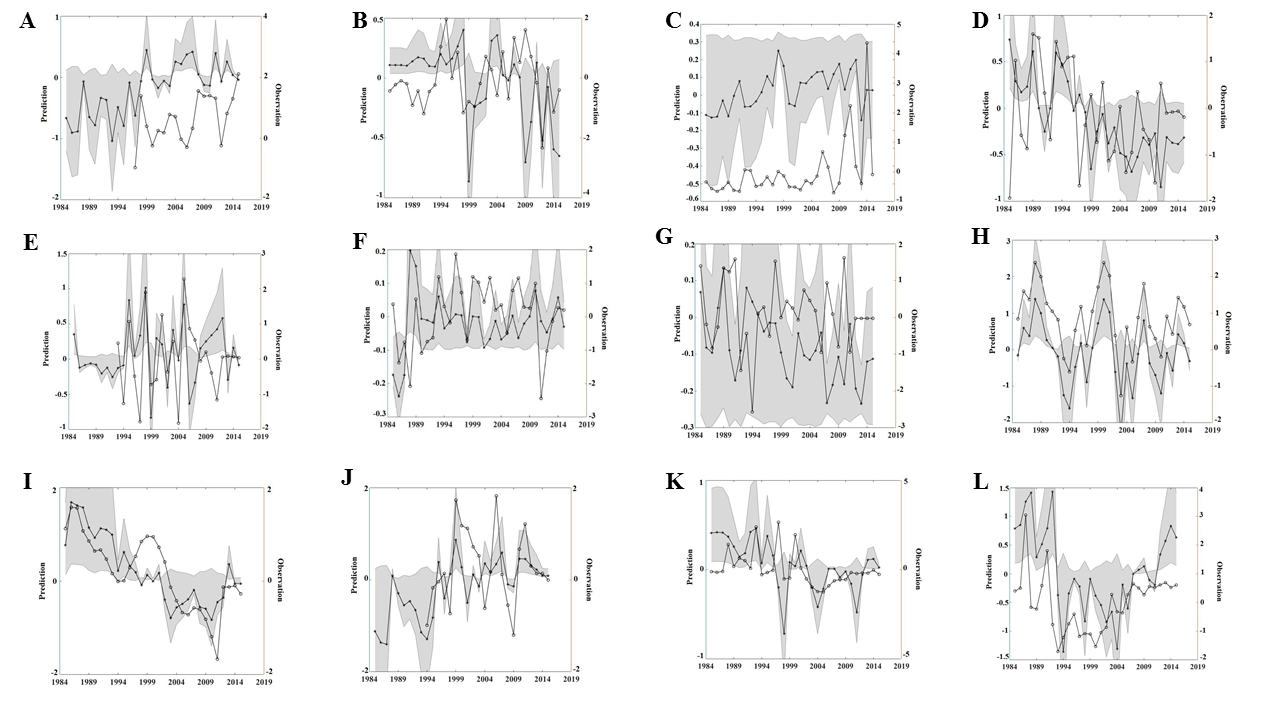

Supplement: S1 Fig — Generated predictions by the DDDBN model. The series marked with stars denote the predictions as opposed to the observed data denoted by circles. 95% confidence intervals report bootstrap prediction’s mean and standard deviation. (TIF) [file pone.0209257.s003.tif]

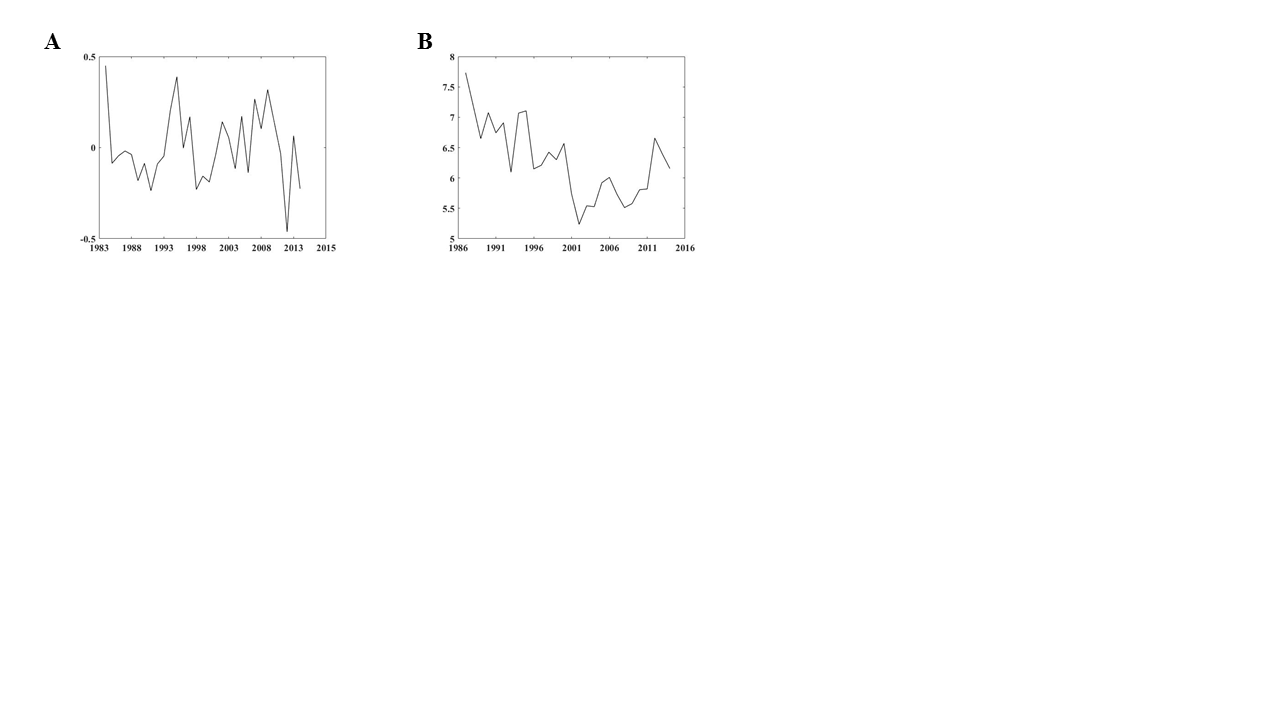

Supplement: S2 Fig — (A) Pink shrimp recruitment deviation. (B) Bottom water dissolved oxygen concentration for the Texas coastal shelf in fall. (TIF) [file pone.0209257.s004.tif]
